# Bactericidal and Antibiofilm Activities of HT-2-1-3, a Vespidae Venom-Derived Antimicrobial Peptide, Against *Streptococcus mutans* UA159

**DOI:** 10.3390/antibiotics15090873

**Published:** 2026-09-07

**Authors:** Yangyang Chen, Yuxiu Ye, Qiurong Wu, Guolian Xu, Tingting Wu, Meiling Tang, Sulan Luo, Yong Wu

**Affiliations:** Guangxi Key Laboratory of Special Biomedicine, School of Medicine, Guangxi University, Nanning 530004, China; 2337030133@st.gxu.edu.cn (Y.C.); 2328403007@st.gxu.edu.cn (Y.Y.); 2337030139@st.gxu.edu.cn (Q.W.); 2337030226@st.gxu.edu.cn (G.X.); 2337030204@st.gxu.edu.cn (T.W.); 2337030129@st.gxu.edu.cn (M.T.)

**Keywords:** antimicrobial peptide, Vespidae venom, *Streptococcus mutans*, dental caries, biosafety

## Abstract

**Background**: Dental caries is a biofilm-associated disease in which *Streptococcus mutans* plays a central role. This study evaluated HT-2-1-3, a Vespidae venom-derived antimicrobial peptide, as a potential topical anti-caries lead. **Methods**: Candidate peptides were synthesized by Fmoc solid-phase peptide synthesis and characterized by RP-HPLC and ESI-MS. Antibacterial activity against *S. mutans* UA159 was assessed using MIC, MBC, growth-curve, and time-kill assays. Membrane damage, antibiofilm activity, cytocompatibility, hemolysis, and preliminary oral safety in mice were further evaluated. **Results**: HT-2-1-3 showed the strongest activity among the tested peptides, with MIC and MBC values of 2 µg/mL. It inhibited bacterial growth in a concentration-dependent manner and achieved complete killing at 4×MIC (8 µg/mL) within 2 h. Mechanistic assays showed rapid membrane depolarization, increased PI uptake, and concentration-dependent release of extracellular DNA and proteins. MD simulations further indicated that HT-2-1-3 entered the model *S. mutans* UA159 membrane at 1857 ns, supporting membrane permeabilization as a central component of its bactericidal activity. Scanning electron microscopy revealed dose-dependent membrane disruption, cell collapse, and rupture. HT-2-1-3 inhibited biofilm formation by approximately 58–63% and eradicated up to 89.0% of mature biofilms. Hemolysis remained below 4% at concentrations up to 64×MIC (128 µg/mL), and mammalian-cell viability exceeded 95% at concentrations up to 16×MIC (32 µg/mL). Repeated oral administration caused no obvious gingival irritation in mice. **Conclusions**: HT-2-1-3 showed strong bactericidal and antibiofilm activity against *S. mutans* UA159 and favorable preliminary biocompatibility. Its mode of action may involve membrane disruption, and it showed favorable preliminary biosafety. These findings support further optimization of HT-2-1-3 as a topical antimicrobial candidate for caries prevention and control.

## 1. Introduction

Dental caries is one of the most common chronic noncommunicable diseases worldwide and remains a substantial public-health and economic burden [1,2]. It develops when dental plaque shifts toward an acidogenic and aciduric microbial community that promotes enamel demineralization. *Streptococcus mutans* is a major cariogenic species because it metabolizes dietary carbohydrates into organic acids, synthesizes extracellular polysaccharides (EPS), and forms structured biofilms on the tooth surface [3,4,5]. The EPS-rich matrix retains acids near enamel and restricts antimicrobial penetration, making mature dental biofilms difficult to remove [5,6].

Plaque control currently depends on mechanical cleaning, fluoride, and topical antiseptics such as chlorhexidine. These approaches are useful in clinical practice, but long-term or nonselective antimicrobial exposure may cause tooth staining, taste disturbance, mucosal irritation, and disruption of oral microbial ecology [7,8]. Concern about antimicrobial resistance has also increased interest in anti-infective agents that act through mechanisms distinct from those of conventional antibiotics [9,10,11]. For anti-caries use, a candidate should reduce viable *S. mutans*, retain activity against biofilm-associated cells, and show acceptable compatibility with host tissues.

Antimicrobial peptides (AMPs) are short host-defense or synthetic peptides that often carry a net positive charge and form amphipathic structures. Many AMPs interact with negatively charged bacterial membranes and disrupt membrane integrity, a mechanism that may lower the risk of classical target-site resistance [9,10,11,12]. In oral research, AMPs and peptide mimics have been explored for inhibiting cariogenic bacteria, preventing biofilm formation, targeting *S. mutans*, and, in some designs, promoting remineralization [13,14,15,16,17,18,19]. However, many reported anti-caries AMPs remain limited by peptide stability, cytotoxicity, production cost, or insufficient testing in mature-biofilm and oral safety models [13,15,16].

*Vespidae* venom contains multiple bioactive peptides and is a useful source for discovering membrane-active antimicrobial molecules. In this study, we screened candidate peptides from an in-house *Vespidae* venom-derived AMP database and identified HT-2-1-3 as the most active peptide against *S. mutans*. We then evaluated its antibacterial activity, killing kinetics, membrane-damaging effects, inhibition of biofilm formation, eradication of mature biofilms, and preliminary in vitro and in vivo safety. We aimed to determine whether HT-2-1-3 remains active against both planktonic *S. mutans* and established biofilms at concentrations tolerated by host cells.

## 2. Results

### 2.1. Peptide Synthesis and Structural Characterization

**Figure 1:**
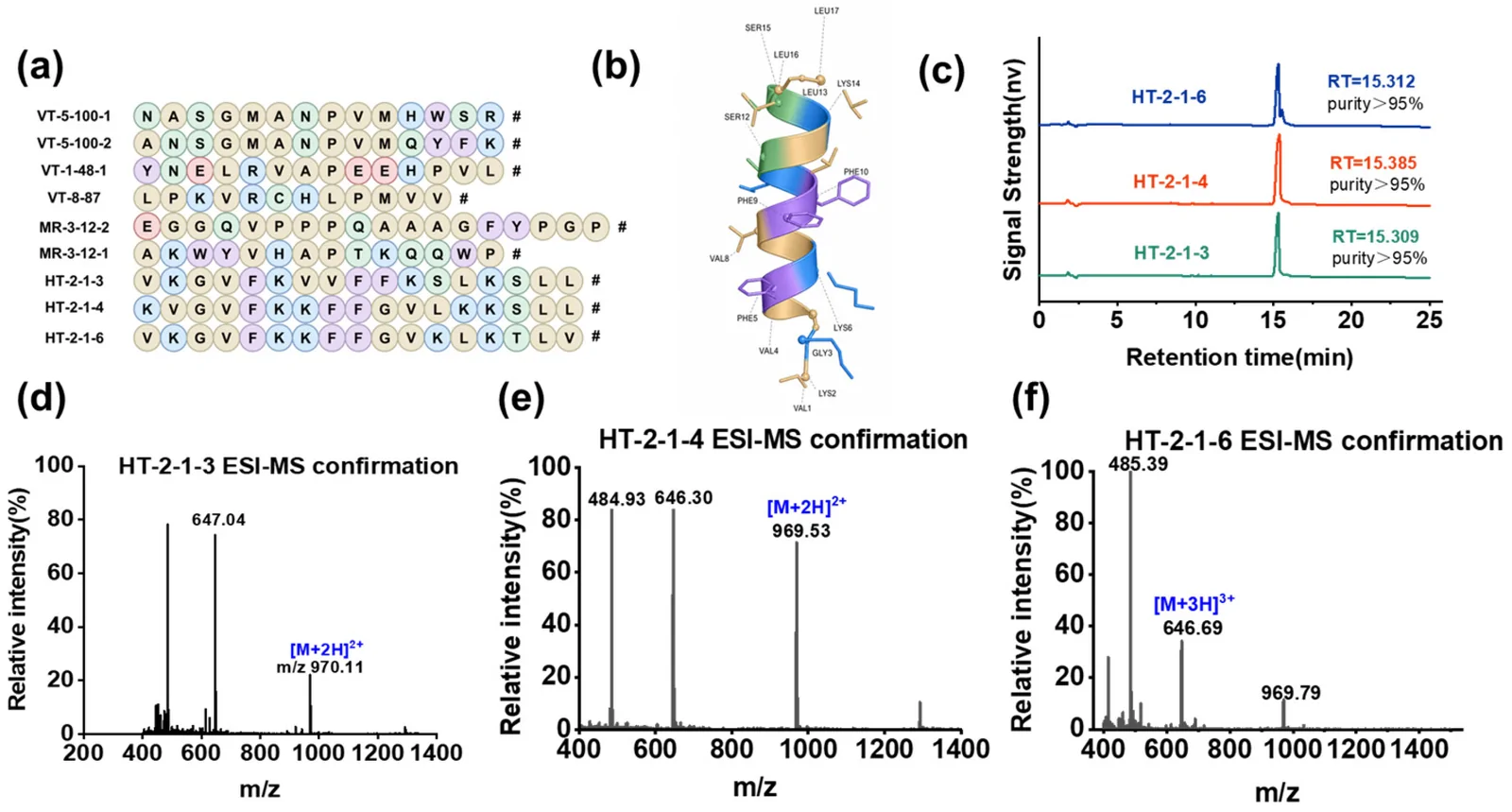
Purification and molecular characterization of the candidate antimicrobial peptides. (**a**) Amino-acid sequence matrix of the candidate peptides. (**b**) Predicted secondary structure of HT-2-1-3. (**c**) RP-HPLC quantitative analysis of HT-2-1-3, HT-2-1-4, and HT-2-1-6. (**d**–**f**) ESI-MS molecular-mass identification of HT-2-1-3, HT-2-1-4, and HT-2-1-6.

Candidate AMPs derived from Vespidae venom were selected from the in-house database and synthesized by Fmoc solid-phase peptide synthesis. After preliminary activity screening, HT-2-1-3, HT-2-1-4, and HT-2-1-6 were selected for detailed characterization (Figure 1a). RP-HPLC analysis showed single symmetric peaks for the three peptides, with retention times of 15.31, 15.39, and 15.31 min, respectively, and integrated peak areas above 95% (Figure 1c). ESI-MS confirmed a measured molecular mass of 1938.46 Da for HT-2-1-3, and the measured masses of HT-2-1-4 and HT-2-1-6 were consistent with their theoretical values (Figure 1d–f). AlphaFold3 predicted that HT-2-1-3 adopts a compact α-helical conformation (Figure 1b), consistent with the structural features of cationic amphipathic AMPs [20]. Sequence alignment showed limited overall identity between HT-2-1-3 and representative Vespidae-derived antimicrobial peptides (17.6–33.3%). EMP-EM1 and EMP-EM2 showed the highest identity (33.3%) (Supplementary Figure S1). These results indicate that HT-2-1-3 has a distinct primary sequence among the selected Vespidae peptide comparators.

### 2.2. Antibacterial Activity Against Streptococcus mutans

**Figure 2:**
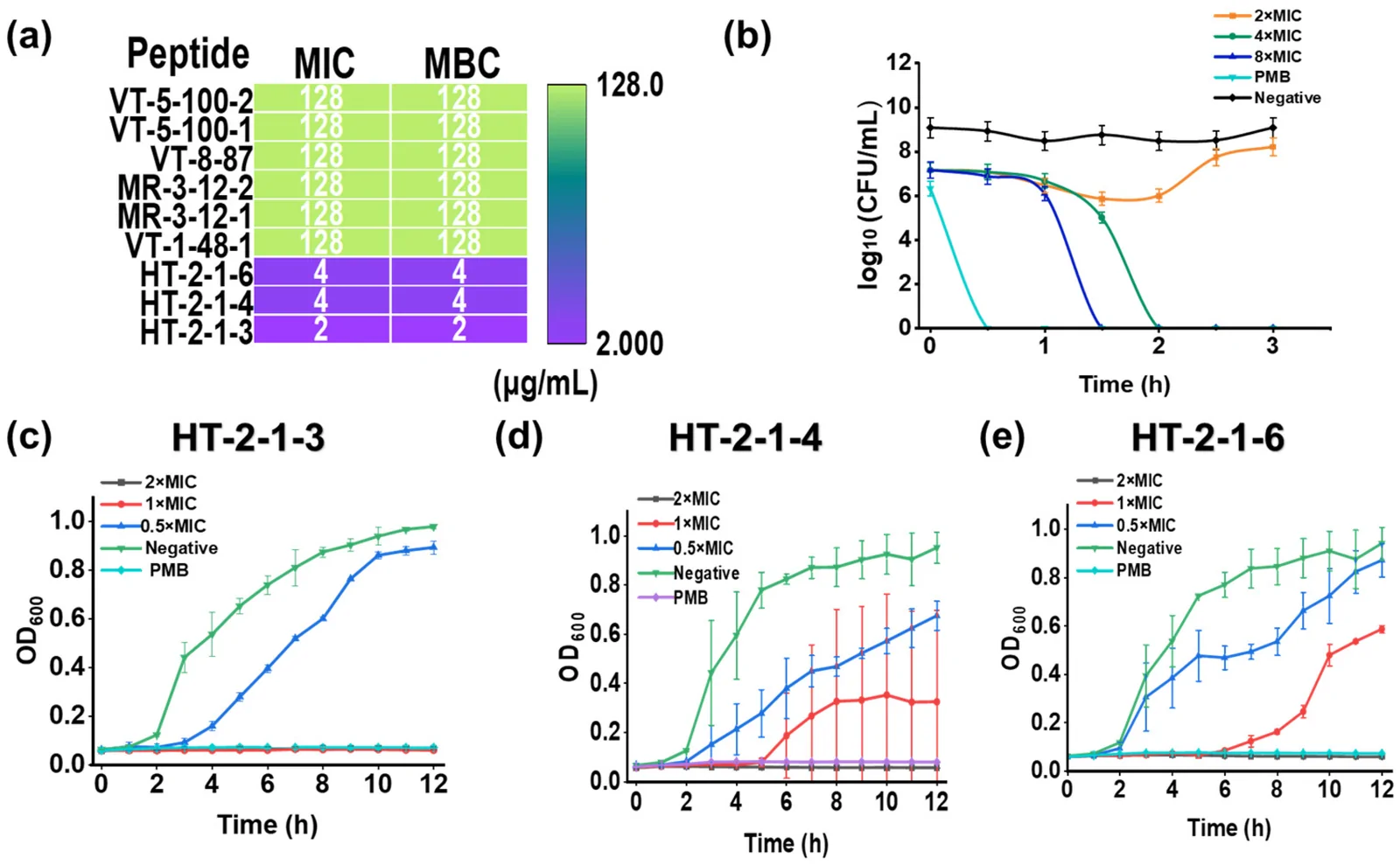
Antibacterial activity and killing kinetics of the antimicrobial peptides against *S. mutans* UA159. (**a**) MIC and MBC values of the different peptides. (**b**) Time-kill curves of HT-2-1-3, expressed as log_10_ CFU/mL over 3 h. (**c**–**e**) OD_600_ growth curves after treatment with different peptide concentrations; polymyxin B served as the positive control. Data are presented as mean ± SD (*n* = 3). The experiment in (**a**) was repeated three times with similar results.

HT-2-1-3, HT-2-1-4, and HT-2-1-6 all inhibited *S. mutans* UA159, with HT-2-1-3 showing the greatest activity (Figure 2a). The MIC and MBC values of HT-2-1-3 were both 2 μg/mL, whereas HT-2-1-4 and HT-2-1-6 each had MIC and MBC values of 4 μg/mL. The MBC/MIC ratio for HT-2-1-3 was 1. Because a ratio ≤4 is generally considered bactericidal, HT-2-1-3 was classified as bactericidal under these assay conditions.

HT-2-1-3 was therefore used for the subsequent mechanistic, antibiofilm, and safety experiments. Polymyxin B was used as the in vitro reference control because it showed measurable activity against *S. mutans* UA159 under the same broth microdilution conditions (MIC = 10 μg/mL), whereas the chlorhexidine MIC exceeded 20 μg/mL. This choice reflects activity in the present assay system and does not imply that polymyxin B is a standard clinical benchmark for caries prevention.

### 2.3. Growth Inhibition and Killing Kinetics

Growth-curve analysis showed concentration-dependent inhibition of *S. mutans* UA159 by the three peptides (Figure 2c–e). At concentrations at or above 2×MIC (4 μg/mL), OD_600_ values remained nearly unchanged over 12 h, indicating effective suppression of bacterial proliferation. At 1×MIC (2 μg/mL), growth was delayed but not fully blocked, indicating partial rather than complete suppression.

Time-kill analysis confirmed rapid concentration-dependent killing by HT-2-1-3 (Figure 2b). At 8×MIC (16 μg/mL), viable counts fell below the detection limit within 1.5 h. At 4×MIC (8 μg/mL), complete killing was achieved within 2 h. Treatment at 2×MIC (4 μg/mL) caused transient suppression followed by partial recovery. These data indicate that sustained clearance of exponential-phase *S. mutans* required exposure above the MIC.

### 2.4. Structural Characterization of HT-2-1-3 by Circular Dichroism

**Figure 3:**
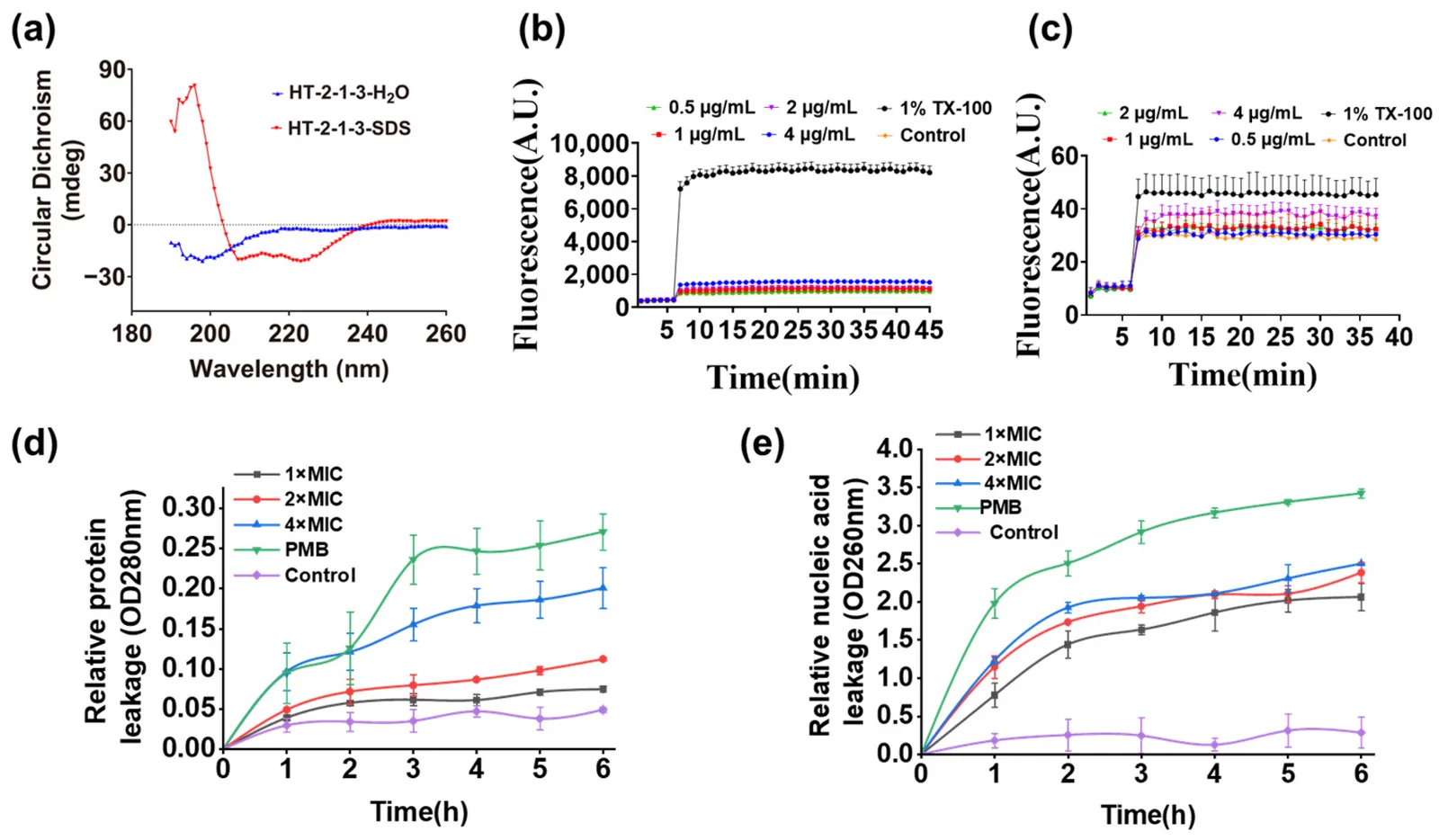
Mechanistic characterization of the antibacterial activity of HT-2-1-3 against *S. mutans* UA159. (**a**) CD spectra of HT-2-1-3 in H_2_O and SDS. (**b**) Membrane depolarization of *S. mutans* UA159 treated with different concentrations of HT-2-1-3. (**c**) PI uptake by *S. mutans* UA159 treated with different concentrations of HT-2-1-3. (**d**,**e**) Leakage of DNA and proteins from *S. mutans* UA159 treated with different concentrations of HT-2-1-3; polymyxin B served as the positive control. Data are presented as mean ± SD (*n* = 3).

The secondary structure of HT-2-1-3 was characterized by circular dichroism (CD) spectroscopy. In H_2_O, the peptide showed a negative ellipticity signal near 200 nm without distinct minima at 208 and 222 nm. This profile indicated a predominantly random-coil conformation. In a sodium dodecyl sulfate (SDS) membrane-mimetic environment, HT-2-1-3 showed distinct negative minima near 208 and 222 nm. A positive maximum at approximately 190–195 nm further indicated a characteristic α-helical conformation (Figure 3a). These results indicate that HT-2-1-3 undergoes a random-coil-to-α-helix transition in a membrane-mimetic environment, which may facilitate bacterial membrane interactions.

### 2.5. Membrane Depolarization and Propidium Iodide Uptake

Membrane depolarization and propidium iodide (PI) uptake assays were performed to assess the effects of HT-2-1-3 on *S. mutans* UA159 membrane function. Fluorescence increased rapidly after HT-2-1-3 treatment and generally rose with peptide concentration, indicating dissipation of the membrane potential (Figure 3b). PI fluorescence also increased rapidly after treatment and remained elevated (Figure 3c), indicating increased membrane permeability. Together, these results show that HT-2-1-3 rapidly disrupts membrane-potential homeostasis and barrier function in *S. mutans* UA159.

### 2.6. HT-2-1-3-Induced Leakage of Intracellular DNA and Proteins

Leakage of intracellular material was then assessed. Extracellular DNA and protein levels increased after HT-2-1-3 treatment and generally rose with peptide concentration. The polymyxin B positive-control group also showed marked leakage of intracellular material (Figure 3d, e). These findings indicate that HT-2-1-3 compromises membrane integrity in *S. mutans* UA159 and releases intracellular components, including DNA and proteins. Together with the membrane-depolarization and PI-uptake results, these data associate HT-2-1-3 activity with disruption of membrane structure and function.

### 2.7. Molecular Dynamics Analysis of HT-2-1-3 Membrane Interactions

**Figure 4:**
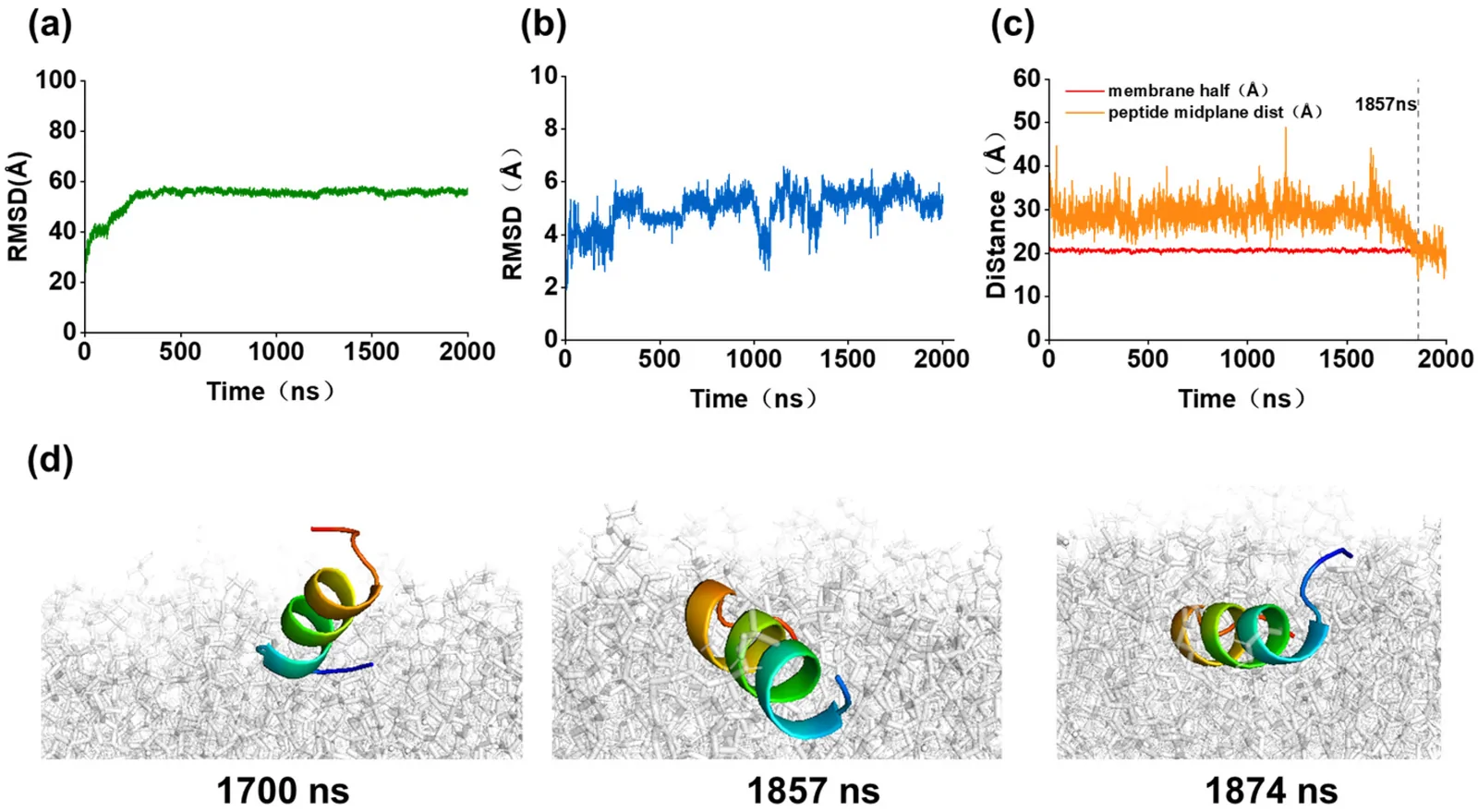
Molecular dynamics (MD) simulation of HT-2-1-3 interacting with an *S. mutans* UA159 model membrane. (**a**) RMSD of the lipid bilayer during the 2000-ns MD simulation. (**b**) RMSD of the peptide backbone during the simulation. (**c**) Penetration kinetics of HT-2-1-3 in the *S. mutans* UA159 membrane. The red line indicates the distance from the membrane surface to the bilayer midplane. The yellow line indicates the distance from the peptide center of mass (COM) to the bilayer midplane. The gray vertical marker indicates when the peptide crossed the membrane surface. (**d**) Positions and orientations of HT-2-1-3 within the *S. mutans* UA159 membrane at 1700, 1857, and 1874 ns.

Molecular dynamics (MD) simulations using a model membrane representative of *S. mutans* UA159 were performed to investigate the interaction of HT-2-1-3 with bacterial membranes. During the 2000-ns simulation, the lipid root-mean-square deviation (RMSD) remained stable (Figure 4a), indicating that the bilayer retained its structural integrity. Peptide-backbone RMSD trajectories showed distinct stabilization behavior between the system (Figure 4b). Notably, the peptide entered the *S. mutans* UA159 membrane at 1857 ns (Figure 4c,d). This event promoted membrane permeabilization and depolarization, consistent with the leakage and fluorescence assays.

### 2.8. Morphological Evidence of Membrane Disruption

**Figure 5:**
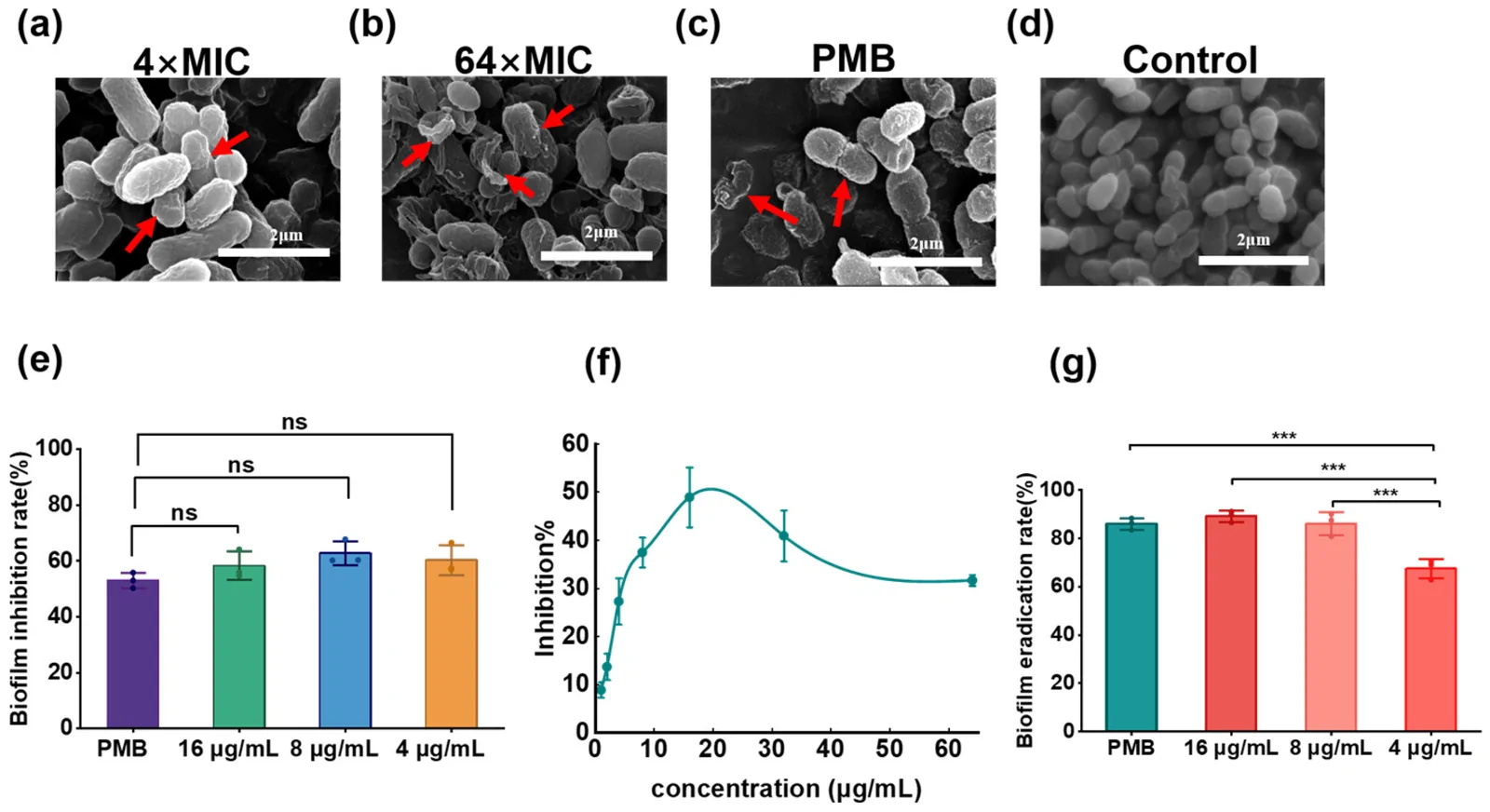
Membrane-disruptive and antibiofilm activities of HT-2-1-3 against *S. mutans* UA159. (**a**–**d**) SEM images after different treatments; red arrows indicate membrane damage, lysis, or morphological disruption; scale bar = 2 μm. *n* = 3. (**e**) Inhibition of biofilm formation by different HT-2-1-3 concentrations and the positive control. (**f**) Dose–response curve of biofilm metabolic activity after HT-2-1-3 treatment. (**g**) Eradication rates of mature biofilms. Data in (**e**–**g**) are presented as mean ± SD, *n* = 3. Values of ns, *p* > 0.05, *** *p* < 0.001 were considered statistically significant.

Scanning electron microscopy revealed clear concentration-dependent morphological damage after HT-2-1-3 treatment (Figure 5a–d). Untreated cells retained a regular coccoid morphology and intact surfaces. At 4×MIC (8 μg/mL), cells showed surface depressions and shallow wrinkling but remained largely intact. Damage became more severe as peptide concentration increased. At 64×MIC (128 μg/mL), many cells collapsed or ruptured, with signs of membrane disruption and intracellular-content leakage. Together with the time-kill results, these changes indicate that membrane damage contributes to rapid killing by HT-2-1-3.

### 2.9. Inhibition and Eradication of S. mutans UA159 Biofilms

Crystal violet staining showed that HT-2-1-3 reduced *S. mutans* UA159 biofilm formation at all tested concentrations (Figure 5e). Biofilm-formation inhibition rates were 60.3%, 62.7%, and 58.2% at 4, 8, and 16 μg/mL, respectively. These values were comparable to the 53.0% inhibition produced by positive-control polymyxin B. MTT assays showed a similar concentration-window effect, with the greatest reduction in metabolic activity at 16 μg/mL and weaker responses at higher concentrations (Figure 5f). The response was therefore non-monotonic, suggesting distinct dose–response patterns for biomass reduction and metabolic inhibition.

HT-2-1-3 also acted against established 48 h mature biofilms (Figure 5g). Eradication rates reached 67.4%, 86.0%, and 89.0% at 4, 8, and 16 μg/mL, respectively. The effect at 16 μg/mL was comparable to polymyxin B (85.9%). The effect at 4 μg/mL was significantly weaker than those at 8 and 16 μg/mL and that of the polymyxin B positive control. These results indicate that HT-2-1-3 suppresses early biofilm formation and reduces established biofilm biomass.

### 2.10. In Vitro and In Vivo Biosafety

**Figure 6:**
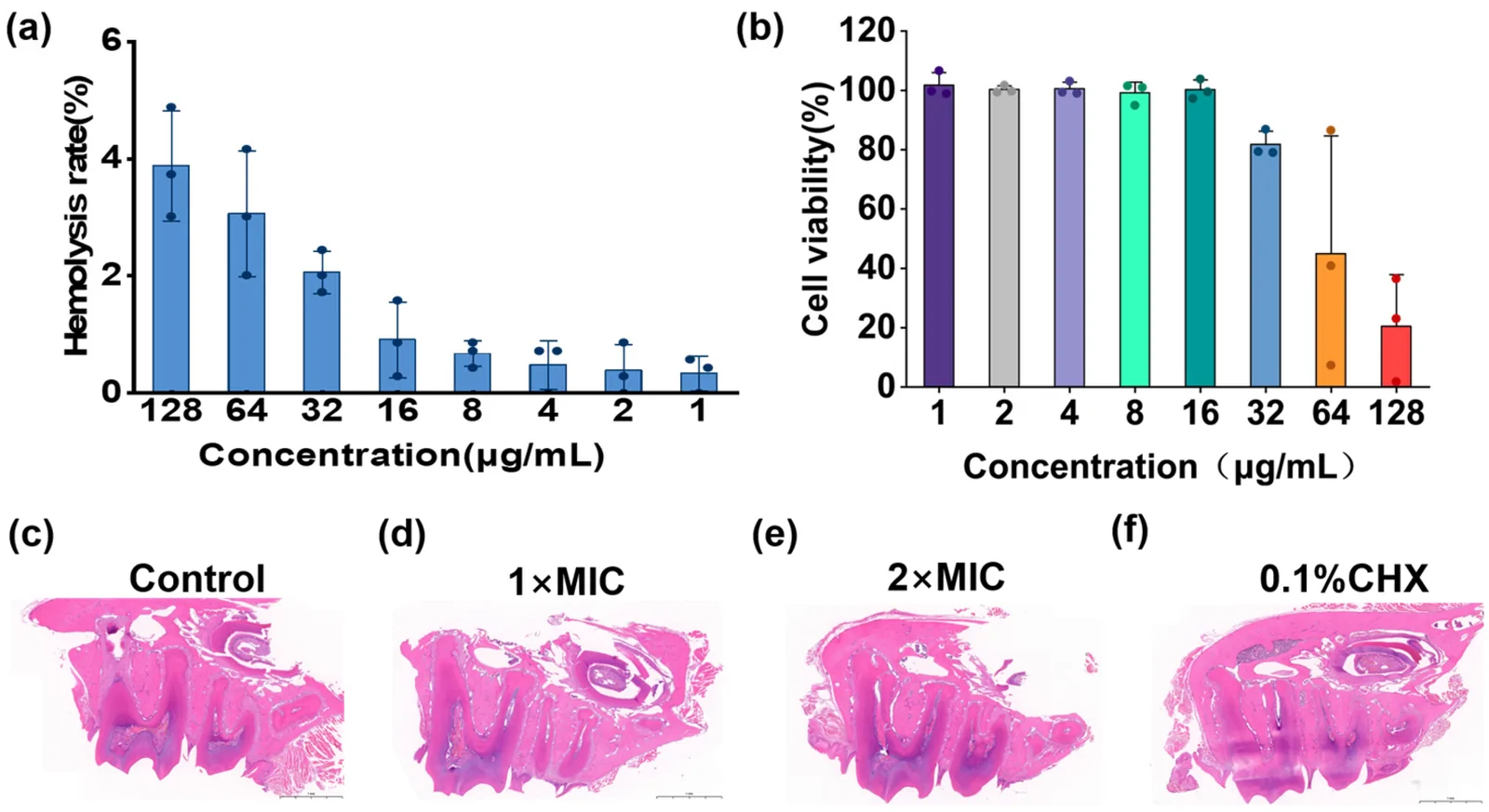
In vitro and in vivo safety evaluation of HT-2-1-3. (**a**) Hemolysis of erythrocytes treated with 1–128 μg/mL HT-2-1-3. (**b**) Cell viability determined by CCK-8 assay after co-incubation with HT-2-1-3. (**c**–**f**) H&E-stained mandibular/oral tissue sections from control, HT-2-1-3-treated groups and 0.1% CHX; scale bar = 1 mm. Data in (**a**,**b**) are presented as mean ± SD, *n* = 3.

HT-2-1-3 showed low host-cell toxicity within its effective antibacterial range (Figure 6). Mean hemolysis remained below 4% at concentrations up to 64×MIC (128 μg/mL), with HC_50_ > 128 μg/mL (Figure 6a). Mammalian-cell viability remained above 95% at concentrations up to 16×MIC (32 μg/mL). Concentration-dependent cytotoxicity appeared at higher concentrations, with a CC_50_ of 101.9 μg/mL and a CC_50_/MIC selectivity index of 51.0 (Figure 6b). In the preliminary mouse oral-caries model, repeated oral administration at MIC (2 μg/mL) and 2×MIC (4 μg/mL) for 30 days caused no obvious local histopathological abnormalities. H&E-stained mandibular sections showed broadly similar tissue architecture across the experimental groups. At low magnification, treated groups showed no obvious mucosal ulceration, extensive tissue necrosis, or marked inflammatory-cell infiltration. These qualitative observations provide preliminary evidence that repeated oral administration caused no overt local tissue injury under the tested conditions. The results indicate a preliminary safety window between antibacterial activity and host-cell toxicity.

## 3. Discussion

This study identified HT-2-1-3 as a Vespidae venom-derived AMP with bactericidal and antibiofilm activity against *S. mutans* UA159. This distinction is important because activity against planktonic *S. mutans* UA159 alone often overestimates anti-caries potential. Reviews of anti-caries peptides suggest that useful candidates should combine bacterial killing, biofilm penetration or disruption, and host compatibility [13,14,15,16]. HT-2-1-3 met several of these early requirements by killing planktonic *S. mutans* UA159, inhibiting biofilm formation, reducing mature biofilm biomass, and showing low hemolysis and limited cytotoxicity within the effective concentration range.

The MIC and MBC of HT-2-1-3 were both 2 μg/mL, indicating bactericidal rather than bacteriostatic activity under the present conditions. Rapid reduction of viable *S. mutans* UA159 may be useful in caries management because it can limit acid production and early biofilm development. Similar activity has been reported for designed oral AMPs such as KR-1, which acted against *S. mutans* UA159, loosened biofilm structure, and killed approximately 90% of bacteria within 5 min under its experimental conditions [21]. Direct comparisons among AMPs should be made cautiously because peptide size, charge, assay medium, inoculum density, and endpoint definitions differ substantially among studies. Even with these limitations, HT-2-1-3 shows in vitro potency comparable to several reported oral AMP candidates. The referenced KR-1 assay used sterile BHI broth and a final bacterial inoculum of 1 × 10^6^ CFU/mL.

The time-kill and SEM data point to membrane disruption as an important mechanism of action. Supra-MIC concentrations of HT-2-1-3 rapidly eliminated viable bacteria, and SEM showed progressive surface deformation, collapse, and rupture. These observations agree with the behavior of many cationic amphipathic AMPs, which bind bacterial envelopes through electrostatic interactions and destabilize membranes through pore-forming or detergent-like mechanisms [10,11,12,22]. The predicted alpha-helical structure of HT-2-1-3 is also consistent with a membrane-active phenotype. Functional assays strengthened this interpretation. HT-2-1-3 caused rapid membrane depolarization, increased PI uptake, and concentration-dependent release of extracellular DNA and proteins. These convergent changes indicate loss of membrane-potential homeostasis, increased permeability, and impaired barrier integrity. MD simulations further showed peptide entry into the model *S. mutans* UA159 bilayer at 1857 ns, consistent with the membrane permeabilization observed experimentally. Together, the morphological, functional, and simulation results support membrane disruption as a central component of HT-2-1-3 bactericidal activity. However, the simulation is model-based and does not establish a complete molecular pathway or exclude additional intracellular effects.

A notable finding is that HT-2-1-3 retained activity against 48 h mature biofilms. Mature *S. mutans* UA159 biofilms are protected by EPS and extracellular DNA, which form a diffusion barrier and increase antimicrobial tolerance [5,6]. HT-2-1-3 eradicated approximately 89% of mature biofilm biomass at 8×MIC (16 μg/mL) and inhibited biofilm formation by approximately 58–63% across the tested range. The MTT assay showed a non-monotonic concentration-response pattern, with maximal inhibition at 16 μg/mL and weaker responses at higher concentrations. The present data cannot determine the cause because peptide aggregation, solubility, and assay-specific effects were not directly evaluated. Confocal live/dead staining, EPS quantification, biofilm-thickness measurement, and three-dimensional reconstruction could clarify whether HT-2-1-3 primarily affects viability, matrix accumulation, adhesion, or biofilm architecture.

Compared with multifunctional anti-caries peptide systems, HT-2-1-3 currently functions mainly as an antimicrobial lead. Recent examples include dual-sensitive antibacterial peptide nanoparticles, gallic-acid–polyphemusin conjugates with antimicrobial and mineralizing properties, and targeted self-assembling peptide hydrogels designed for pathogen control and enamel repair [23,24,25]. HT-2-1-3 is simpler than these systems, but its value lies in its strong anti-*S. mutans* UA159 activity, mature-biofilm reduction, and low host-cell toxicity within the active concentration range. Formulation studies could improve oral retention, proteolytic stability, pH responsiveness, or tooth-surface affinity, all of which would be needed before practical topical oral use is considered.

Within the antibacterial concentration range, HT-2-1-3 showed low hemolysis and preserved mammalian-cell viability. It caused less than 4% hemolysis even at 64×MIC (128 μg/mL) and maintained high mammalian-cell viability at concentrations up to 16×MIC (32 μg/mL). Repeated oral administration in mice did not produce obvious gingival irritation. These results suggest a preliminary therapeutic window between antibacterial efficacy and host-cell toxicity. However, the oral cavity includes conditions not fully reproduced in this study, such as saliva flow, proteolytic enzymes, acidic episodes, epithelial barriers, and continuous interaction with commensal microorganisms [13,16,26]. Additional safety testing should include oral epithelial cells, gingival fibroblasts, repeated-dose histopathology, and inflammatory-marker assessment.

This study has several limitations. The antibacterial assays were performed mainly with the standard strain *S. mutans* UA159, so activity against clinical isolates remains to be confirmed. The experiments also did not model multispecies dental plaque containing other *S. mutans* strains, lactobacilli, Candida albicans, and health-associated oral streptococci. The effect of HT-2-1-3 on commensal oral bacteria remains unknown, which is important because nonselective antimicrobial pressure may disturb oral ecological balance [14,16]. In addition, the stability of HT-2-1-3 under simulated salivary conditions was not assessed. Salivary proteases, ionic composition, pH fluctuations, and dilution may affect peptide integrity and activity. Therefore, stability and residual antibacterial activity in simulated saliva should be investigated before practical oral use is considered.

Finally, because the mouse study included only three animals per group and provided mainly qualitative observations, the in vivo findings should be considered preliminary. Before confirmatory in vivo efficacy studies, HT-2-1-3 retention, antibiofilm activity, and potential effects on enamel surfaces should be evaluated in saliva-pretreated ex vivo bovine-tooth biofilm models. The current results provide initial evidence of local oral tolerability but do not establish definitive safety or anti-caries efficacy. Future studies should use adequately powered cohorts and prespecified quantitative endpoints, including lesion severity, enamel demineralization, histopathology, inflammatory markers, and oral-microbiome changes.

Taken together, HT-2-1-3 should be viewed as a natural-product-derived antimicrobial lead rather than a finished anti-caries therapy. Its bactericidal potency, membrane-damaging activity, mature-biofilm reduction, and preliminary biocompatibility justify further testing. The next stage should focus on validation against clinical isolates, multispecies biofilm models, oral microbiome selectivity, resistance-development assays, peptide stability, and local delivery systems suitable for short-contact oral application.

## 4. Materials and Methods

### 4.1. Peptide Selection, Synthesis, Purification, and Structural Prediction

Candidate peptides were selected from an in-house *Vespidae* venom-derived AMP database using a workflow based on physicochemical descriptors and predicted antimicrobial potential. The selected peptides included HT-2-1-3, HT-2-1-4, HT-2-1-6, VT-1-48-1, MR-3-12-1, MR-3-12-2, VT-8-87, VT-5-100-1, and VT-5-100-2. Peptides were synthesized using CEM Liberty Blue microwave-assisted Fmoc solid-phase peptide synthesis, with Rink amide resin as the solid support (Liberty Blue, CEM, USA) [27]. Fmoc groups were removed with 20% piperidine in DMF, and Fmoc-protected amino acids were coupled stepwise from the C-terminus to the N-terminus using the Oxyma activation system. Peptides were cleaved and side chains deprotected with a trifluoroacetic acid/water/triisopropylsilane cocktail, precipitated with cold diethyl ether, centrifuged, lyophilized, and purified by preparative RP-HPLC. Fractions with purity above 95% were collected and lyophilized, and molecular masses were confirmed by positive-ion ESI-MS (Figures S2 and S3). The three-dimensional structure of HT-2-1-3 was predicted using AlphaFold3, and the model with the highest confidence was selected for visualization and secondary-structure analysis [20].

### 4.2. Bacterial Strain and Culture Conditions

*S. mutans* UA159 was preserved in 25% glycerol at −80 °C. Before experiments, bacteria were streaked onto brain heart infusion (BHI) agar plates and incubated at 37 °C under 5% CO_2_ for 24 h. Single colonies were inoculated into BHI broth, cultured overnight under the same conditions, and diluted to the required concentration for each assay.

### 4.3. MIC and MBC Determination, Growth-Curve Analysis, and Time-Kill Assays

MIC and MBC were determined by broth microdilution with reference to CLSI M07 and adjusted for oral-streptococcal growth characteristics [28]. Peptides were serially two-fold diluted in BHI broth to final concentrations of 0.5–64 μg/mL. The dilutions were mixed with bacterial suspension to obtain a final inoculum of 1 × 10^6^ CFU/mL. Polymyxin B (10 μg/mL) served as the positive control, and BHI broth served as the blank control. After incubation at 37 °C under 5% CO_2_ for 24 h, the lowest concentration without visible turbidity was defined as the MIC. Aliquots from non-turbid wells were plated on BHI agar. The lowest concentration causing a ≥99.9% reduction in viable colonies was defined as the MBC.

Growth curves were generated by measuring OD_600_ every hour for 12 h using an automated microplate reader. For time-kill assays, exponential-phase *S. mutans* UA159 was incubated with HT-2-1-3 at 1×MIC, 2×MIC, 4×MIC, and 8×MIC (2–16 μg/mL). Samples were collected at 0, 0.5, 1, 1.5, 2, 2.5, and 3 h. Samples were serially diluted in PBS, plated on BHI agar, and counted after incubation.

### 4.4. Circular Dichroism Spectroscopy

Peptide secondary structure was analyzed by CD spectroscopy (Chirascan, Applied Photophysics, UK). in aqueous solution (deionized water) and 30 mM SDS. CD spectra were recorded over the wavelength range of 190–260 nm using peptide solutions at a final concentration of 100 μM. Solution-phase CD measurements and secondary-structure analyses followed previously reported methods [29,30]. Spectra of buffer alone and SDS alone served as blanks and were subtracted from all peptide measurements.

### 4.5. Membrane Depolarization and Propidium Iodide Uptake Assays

Inner-membrane integrity was evaluated using a propidium iodide (PI) uptake assay. Mid-log-phase bacterial cultures were harvested by centrifugation at 5000× *g* for 10 min at 4 °C. The pellets were resuspended in HEPES buffer (pH 7.4) containing 20 mM glucose to an OD_600_ of 0.5. Bacterial suspension (50 μL) was mixed with 50 μL of peptide solution in a black 96-well plate and incubated at 37 °C for 15 min. PI solution (10 μL; 50 μg/mL) was then added. Fluorescence was monitored continuously for 30 min using a SpectraMax M2/M2e microplate reader(SpectraMax M2/M2e, Molecular Devices, USA). Excitation and emission wavelengths were 535 and 617 nm, respectively. All experiments were performed in triplicate.

The cytoplasmic membrane potential of *S. mutans* was measured using 3,3′-dipropylthiadicarbocyanine iodide [DiSC_3_(5)]. Mid-log-phase bacteria were collected by centrifugation at 5000× *g* for 10 min at 4 °C. The pellets were resuspended in HEPES buffer containing 20 mM glucose and 90 mM KCl to an OD_600_ of 0.5. Bacterial suspension (50 μL) was mixed with 10 μL of 10 μM DiSC_3_(5) in a black 96-well plate. Baseline fluorescence was recorded immediately for 6 min. Peptide solution (50 μL) was then added, and the plate was incubated in the dark at 37 °C for 30 min. Fluorescence was recorded continuously for a further 45 min. Excitation and emission wavelengths were 622 and 673 nm, respectively, on a SpectraMax M2/M2e microplate reader(SpectraMax M2/M2e, Molecular Devices, USA). All assays were performed in three independent experiments.

### 4.6. Extracellular DNA and Protein Leakage Assays

Bacterial suspensions (2 × 10^8^ CFU/mL) were mixed with equal volumes of peptide solutions at 1×, 2×, and 4×MIC and incubated at 37 °C. At the indicated time points, samples were centrifuged, and the supernatants were analyzed using a DeNovix DS-11 nanovolume spectrophotometer (DeNovix DS-11, DeNovix, USA). Absorbance was recorded at 260 and 280 nm. Untreated bacterial suspensions served as the negative control. Each assay was performed in duplicate and repeated in three independent experiments.

### 4.7. Molecular Dynamics Simulations of Peptide-Membrane Interactions

The C terminus of HT-2-1-3 was amidated using CHARMM-GUI [31,32]. A lipid bilayer mimicking the *S. mutans* UA159 membrane was constructed with a POPE:POPG ratio of 3:1. The peptide structure was predicted using AlphaFold3, and the MD system was built in CHARMM-GUI. The first principal axis of HT-2-1-3 was oriented perpendicular to the z axis, 35 Å above the membrane surface. The peptide-membrane system was solvated with the three-point transferable intermolecular potential (TIP3P) water model in a rectangular periodic box. Water layers 22.5 Å thick were retained above and below the system. Na^+^ and Cl^−^ were added to 150 mM while maintaining overall charge neutrality.

MD simulations were performed using GROMACS 2024.3 and the CHARMM36m protein force field implemented through CHARMM-GUI. The system temperature and pressure were set to 310.15 K and 1 atm, respectively. During pre-equilibration, 5000 steps of steepest-descent minimization were performed over a total of 1857 ps. Minimization was considered converged when the system energy fell below 1000 kJ/mol. The system was then gradually heated and pressurized to the target conditions. Production simulations were conducted in the isothermal-isobaric (NPT) ensemble for 2000 ns, and configurations were saved every 100 ps.

During pre-equilibration and production, temperature was maintained with a velocity-rescaling thermostat using a 1-ps time constant. Pressure was maintained with a C-rescaling barostat using a 5-ps time constant.

### 4.8. Scanning Electron Microscopy

Exponential-phase *S. mutans* UA159 cells were treated with HT-2-1-3 at 4×MIC (8 μg/mL) to 64×MIC (128 μg/mL) for 2 h. Cells were collected by centrifugation and fixed overnight in 2.5% glutaraldehyde at 4 °C. After graded ethanol dehydration, tert-butanol freeze-drying, mounting on aluminum stubs, and gold sputter coating, bacterial morphology was examined by scanning electron microscopy at 20 kV.

### 4.9. Biofilm-Formation Inhibition and Mature-Biofilm Eradication Assays

Crystal violet staining was used to evaluate inhibition of biofilm formation and eradication of mature biofilms [33]. For formation-inhibition assays, exponential-phase *S. mutans* UA159 was inoculated into 24-well plates with HT-2-1-3 at 2×MIC (4 μg/mL) to 8×MIC (16 μg/mL). Plates were incubated at 37 °C under 5% CO_2_ for 24 h. Wells were washed with PBS, fixed with methanol, stained with 0.1% crystal violet, and destained with 95% ethanol. Absorbance was measured at 590 nm.

For mature-biofilm eradication, 48 h biofilms were established, washed with PBS, and treated with HT-2-1-3 at 2×MIC (4 μg/mL) to 8×MIC (16 μg/mL) for 24 h before quantification. Biofilm metabolic activity was evaluated using the MTT assay. After biofilm formation in 96-well plates, HT-2-1-3 at 0.5–64×MIC (1-128 μg/mL) was added for 24 h. MTT solution was then added and incubated in the dark for 4 h. Formazan crystals were dissolved in DMSO, and absorbance was measured at 490 nm.

### 4.10. Hemolysis and Cell-Viability Assays

The in vitro biosafety of HT-2-1-3 was evaluated by mouse erythrocyte hemolysis and CCK-8 assays. Fresh mouse erythrocytes were washed with sterile PBS, prepared as an 8% (*v*/*v*) suspension, mixed with an equal volume of HT-2-1-3 solution, incubated at 37 °C for 1 h, and centrifuged. Supernatant absorbance was measured at 540 nm, with 1% Triton X-100 as the 100% hemolysis control.

For cytotoxicity evaluation, mouse macrophages were seeded in 96-well plates at 4 × 10^4^ cells/well and allowed to adhere for 24 h. Cells were then treated with complete DMEM containing HT-2-1-3 at 0.5–64×MIC for 24 h. CCK-8 reagent was added, plates were incubated for 1 h, and absorbance was measured at 450 nm.

### 4.11. Preliminary Mouse Oral Safety Assessment

Four-week-old female ICR mice were randomly assigned to four groups (*n* = 3 per group): a saline negative-control group, a 0.1% chlorhexidine (CHX) positive-control group, a low-concentration HT-2-1-3 group (1×MIC, 2 μg/mL), and a high-concentration HT-2-1-3 group (2×MIC, 4 μg/mL). Beginning 7 days before treatment, the oral cavities of the mice were inoculated with an *S. mutans* UA159 suspension at 1 × 10^9^ CFU/mL. Each mouse received 100 μL of the bacterial suspension by topical oral application once daily for 7 consecutive days.

During the 30-day experimental period, all groups were provided with a high-sugar pelleted cariogenic diet (NIH 2000 cariogenic diet) and drinking water containing 5% sucrose. Saline, 0.1% CHX, or HT-2-1-3 was administered once daily by topical oral application at a volume of 100 μL per mouse. General health was monitored daily, and the oral mucosa was examined for visible irritation, ulceration, or other local adverse effects.

At the end of the experiment, the mice were euthanized. The mandibles were collected, fixed in 4% paraformaldehyde for 24 h, and subsequently processed for hematoxylin and eosin (H&E) staining.

### 4.12. Statistical Analysis

Data are presented as mean +/− standard deviation (SD). All in vitro experiments were performed with at least three independent biological replicates. Between-group comparisons were performed by one-way analysis of variance followed by Tukey’s multiple-comparison test using Origin 2026 Pro. Values of ns, *p* > 0.05, * *p* < 0.05, ** *p* < 0.01, and *** *p* < 0.001 were considered statistically significant.

## 5. Conclusions

HT-2-1-3, a Vespidae venom-derived antimicrobial peptide, showed bactericidal and antibiofilm activity against *S. mutans* UA159 in the present in vitro assays. It rapidly reduced bacterial viability, inhibited biofilm formation, and reduced mature-biofilm biomass at concentrations that showed low hemolysis and limited cytotoxicity. Membrane depolarization, propidium iodide uptake, extracellular DNA and protein leakage, scanning electron microscopy changes, and molecular dynamics simulations together support membrane disruption as a major contributor to its antibacterial activity. In a preliminary mouse oral safety assessment, repeated topical administration caused no overt local tissue injury. These findings identify HT-2-1-3 as a promising lead for topical caries-control strategies. Its translational potential should be evaluated further using clinical isolates and multispecies oral biofilms, with assessment of effects on commensal microbiota, resistance development, stability in saliva and acidic environments, and formulations that improve oral retention.

## Data Availability

The data generated or analyzed during this study are included in this article and its Supplementary Materials. Additional raw data are available from the corresponding author upon reasonable request.

## Supplementary Materials

The following supporting information can be downloaded at: https://www.mdpi.com/article/10.3390/antibiotics15090873/s1, Figure S1: Amino acid sequence alignment of HT-2-1-3, EMP-EM1, EMP-EM2, Mastoparan-V1, Mastoparan-C, Crabrolin, and Mastoparan-J. Figure S2: RP-HPLC quantitative analysis of VT-1-48-1, VT-8-87, MR-3-12-1, VT5-100-1, MR-3-12-2 and VT-5-100-2. Figure S3: ESI-MS molecular mass identification of VT-1-48-1, VT-8-87, MR-3-12-1, VT5-100-1, MR-3-12-2 and VT-5-100-2.

## Abbreviations

The following abbreviations are used in this manuscript:

*S. mutans*	*Streptococcus mutans*
RP-HPLC	reversed-phase high-performance liquid chromatography
ESI-MS	electrospray ionization mass spectrometry
MIC	minimum inhibitory concentration
MBC	minimum bactericidal concentration
AMPs	antimicrobial peptides
SEM	scanning electron microscopy
DMF	N, N-dimethylformamide
BHI	brain heart infusion
DMSO	dimethyl sulfoxide
CD	circular dichroism
SDS	sodium dodecyl sulfate
PMB	polymyxin B
CHX	chlorhexidine
