# Supplementary material for: Bactericidal and Antibiofilm Activities of HT-2-1-3, a Vespidae Venom-Derived Antimicrobial Peptide, Against Streptococcus mutans UA159
Source: Antibiotics (Basel). 2026 Sep 7;15(9):873. doi: 10.3390/antibiotics15090873 (PMC13603369; doi:10.3390/antibiotics15090873)
Supplement: Supplementary file 1 [file antibiotics-15-00873-s001.zip › antibiotics-4485196-supplementary.pdf]

Supporting Information

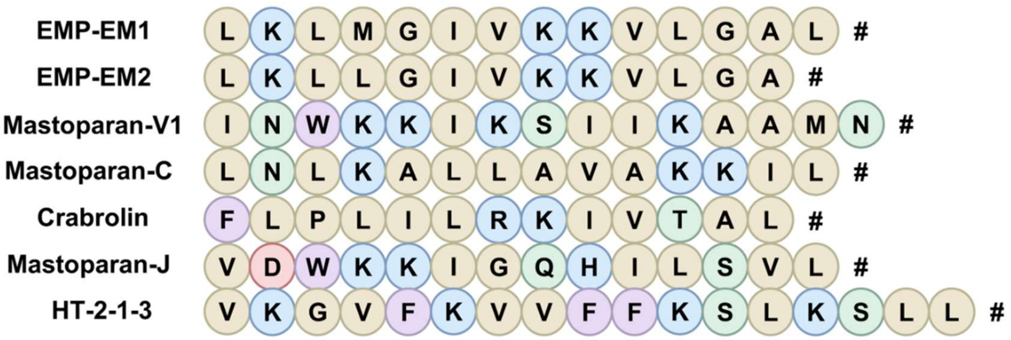

Figure S1. Amino acid sequence alignment of HT-2-1-3, EMP-EM1, EMP-EM2, Mastoparan-V1, Mastoparan-C, Crabrolin, and Mastoparan-J.

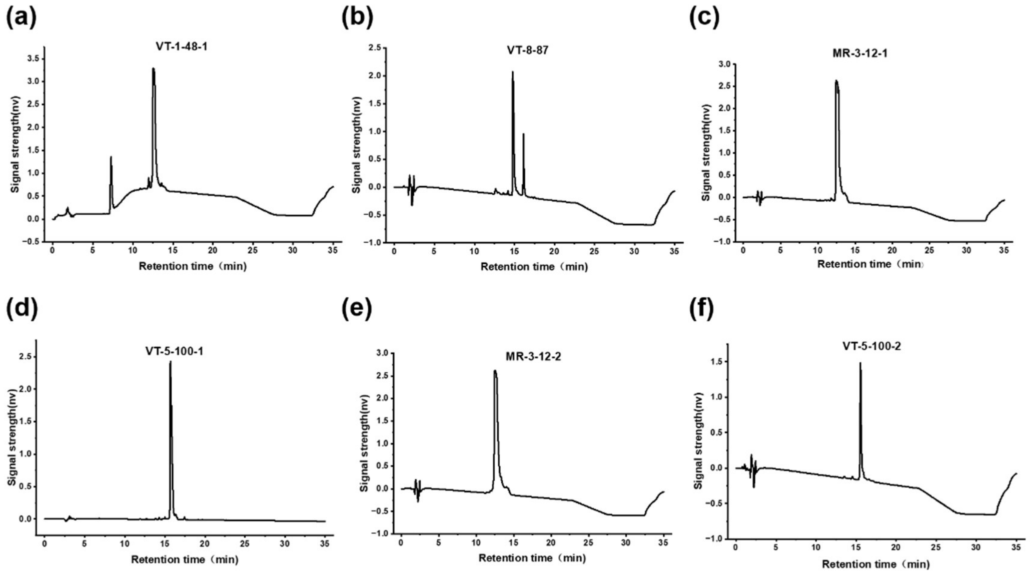

Figure S2. RP-HPLC quantitative analysis of VT-1-48-1, VT-8-87, MR-3-12-1, VT5-100-1, MR-3-12-2 and VT-5-100-2.

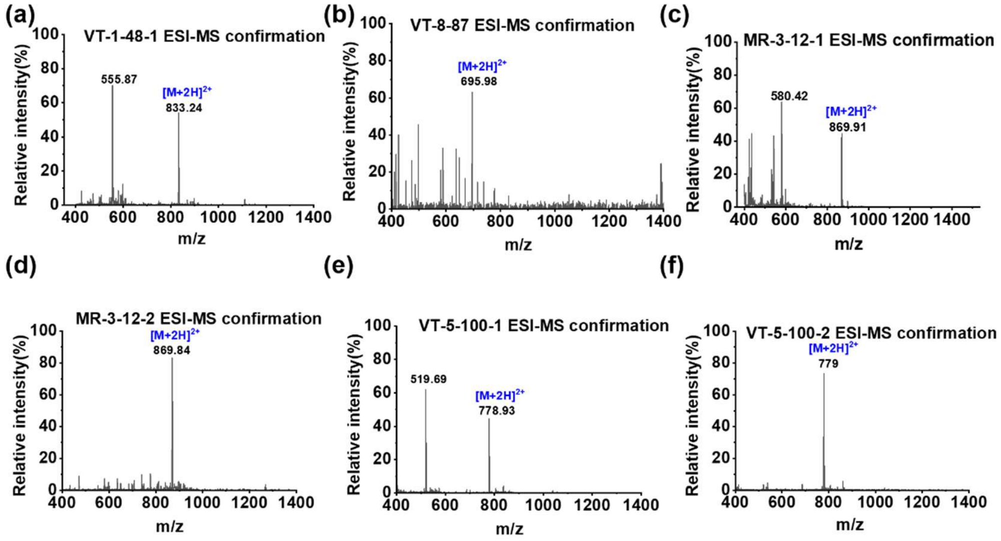

Figure S3.ESI-MS molecular mass identification of VT-1-48-1, VT-8-87, MR-3-12-1, VT5-100-1, MR-3-12-2 and VT-5-100-2.
